# From theory to experimental design—Quantifying a trait-based theory of predator-prey dynamics

**DOI:** 10.1371/journal.pone.0195919

**Published:** 2018-04-25

**Authors:** A. N. Laubmeier, Kate Wootton, J. E. Banks, Riccardo Bommarco, Alva Curtsdotter, Tomas Jonsson, Tomas Roslin, H. T. Banks

**Affiliations:** 1 Center for Research in Scientific Computation, North Carolina State University, Raleigh, NC, United States of America; 2 Department of Ecology, Swedish University of Agricultural Sciences, Uppsala, Sweden; 3 Undergraduate Research Opportunities Center, California State University, Monterey Bay, Seaside, CA, United States of America; University of Sydney, AUSTRALIA

## Abstract

Successfully applying theoretical models to natural communities and predicting ecosystem behavior under changing conditions is the backbone of predictive ecology. However, the experiments required to test these models are dictated by practical constraints, and models are often opportunistically validated against data for which they were never intended. Alternatively, we can inform and improve experimental design by an in-depth pre-experimental analysis of the model, generating experiments better targeted at testing the validity of a theory. Here, we describe this process for a specific experiment. Starting from food web ecological theory, we formulate a model and design an experiment to optimally test the validity of the theory, supplementing traditional design considerations with model analysis. The experiment itself will be run and described in a separate paper. The theory we test is that trophic population dynamics are dictated by species traits, and we study this in a community of terrestrial arthropods. We depart from the Allometric Trophic Network (ATN) model and hypothesize that including habitat use, in addition to body mass, is necessary to better model trophic interactions. We therefore formulate new terms which account for micro-habitat use as well as intra- and interspecific interference in the ATN model. We design an experiment and an effective sampling regime to test this model and the underlying assumptions about the traits dominating trophic interactions. We arrive at a detailed sampling protocol to maximize information content in the empirical data obtained from the experiment and, relying on theoretical analysis of the proposed model, explore potential shortcomings of our design. Consequently, since this is a “pre-experimental” exercise aimed at improving the links between hypothesis formulation, model construction, experimental design and data collection, we hasten to publish our findings before analyzing data from the actual experiment, thus setting the stage for strong inference.

## Introduction

### Quantifying theory—A challenging process

Models are abstractions of reality, distilling presumed relationships. In empirical science, model credibility is established by predicting outcomes observable in real systems. This is the ultimate test of a model: the better it holds up to tests which should easily prove it wrong, the more confidently we can conclude that it reveals something useful about nature and incorporates important mechanisms dictating system behavior. While this is a fundamental principle of modern science [1], the steps between general theory, formulation of an explicit model, and application of this model to predict the outcome of an empirical experiment are typically challenging. Ecological data tend to be both messy and expensive to obtain. Thus, by intimately linking the development of the model to the design of the experiment testing the model, we are able to ensure that we optimize the amount of useful information collected relative to the amount of work required to collect it. To obtain maximal data for resources invested, we need to cunningly design experiments and sample only as much as necessary. By gauging our approach in advance of performing the actual experiment, we obtain rigor in our approach, minimize sources of uncontrolled variation, and sample only as much as needed. Designing our experiments to target clear-cut predictions and estimating well-defined quantities also increases the conclusiveness of the test. Strong inference requires that experiments are designed specifically to test and distinguish among *a priori* hypotheses [2]. To encourage and support this distinction, some fields, such as psychology and neurology, have started promoting pre-registration of research [3–5]. In addition to encouraging clear *a priori* hypotheses, pre-registration has the advantage of enabling researchers to critically examine their hypotheses and plans before beginning their research and providing the chance to receive feedback and improve experimental design [3]. Here, we carefully perform the steps between ecological theory and experimental testing of that theory, before conducting the actual experiment. The analysis we present is intrinsically linked to our ecological hypotheses and associated model, but we advocate for the application of this same approach to other study systems. The experiment itself, following the design we arrive at here, will be presented in a subsequent publication.

### A specific theory in need of testing

Predicting how ecological communities will respond to changes in abundance or composition is a high priority for ecologists and conservationists, requiring the use of models describing the communities and their driving mechanisms. However, it is well understood that “all models are wrong, but some models are useful” [6]. We consider a model useful if it accurately captures the dynamics it is supposed to describe along with a significant portion of the most important mechanisms. Developing such a model requires (i) creating the model with what are thought to be the most important mechanisms, based on the information available, and (ii) exploring that model to determine if it behaves as one would expect and gives robust results. This step includes asking how sensitive the model is to parameter values and whether or not the chosen model formulation is useful. The final step (iii) is testing the model performance and parameterizing the resultant model using empirical data. Establishing a useful predictive model usually requires a number of rounds of (i)-(iii).

We begin with the theory of why and how species interact to create an ecological community, and specifically that these interactions are dictated by species traits. To make accurate predictions about a particular ecological community requires an understanding of its underlying interaction structure and governing dynamics. Empirically obtaining this information is difficult, possibly requiring multiple observations of interactions between predator and prey to determine interaction frequencies as well as experiments to determine the impact of changes to species abundances or presence. There is, however, a growing body of research indicating that species interactions are influenced by their traits, and that these traits can be used, at least to some extent, to predict interaction strength and resulting population dynamics [7, 8]. For example, larger predators have higher metabolic demands and can capture larger prey, while prey can use defenses such as a shell or hard integument to protect themselves from predators [9–12]. These traits show promise in predicting community structure and dynamics as well as, ultimately, how communities respond to changing environmental conditions and species compositions [12–14].

Predictions based on species traits can then be used to manage communities subject to environmental change or to manipulate communities to achieve a particular outcome, e.g. to manage for enhanced ecosystem services such as pest control by natural predators [15, 16]. Predators require traits that increase their ability to locate, capture, and consume their prey, while prey require traits that decrease their chances of being consumed [7]. It is, therefore, the match between predator and prey traits that determines the strength of the trophic interaction between them. Understanding and quantifying the nature of this relationship between traits and trophic interactions gives the two-fold advantage of understanding of the mechanisms behind the interactions as well as potentially reconstructing a food web with less empirical information. This allows for the development of models that predict community population dynamics.

The utility of traits in predicting food web dynamics lies in knowing which few, ideally easily measurable traits, are most important for governing interactions. Body size is a prime candidate as it directly relates to the metabolism (and thus ecophysiology) of a species [17] and determines a number of other characteristics and behavior, such as diet generality [18] and range of movement [19]. Additionally, body mass is well-studied as an important trait in governing food web dynamics [20–23] and the Allometric Trophic Network (ATN) model in particular has been successfully applied to predict the outcome of trophic interactions in microcosm studies of terrestrial arthropods [8, 24]. We therefore begin with the ATN model as used in [8], which models intraspecific competition and can allow for intraguild predation, but which does not support explicit interspecific predator interference. The interplay of different predator species is particularly important in determining trophic interactions, as communities of predators can have both complementary and adversarial effects on one another. In order to capture the complex effect this might have on herbivore populations [25–28], we modify the ATN model to accommodate intraguild interactions beyond predation.

Although body size can explain a large proportion of interactions and is well-studied in a range of systems [8, 12, 29–31], in many cases it is either unimportant or acts primarily as a filter, such that two individuals can, but do not necessarily, have a trophic interaction if they are within a relatively broad size spectrum [32]. Within that size spectrum, other traits may be required to explain why an interaction occurs, and efforts have been made to inform models with additional traits [18, 22, 33]. The problem lies in determining which other traits to include. There is little understanding of how different traits might affect interaction strength and resulting population dynamics (but see [13, 15, 34]). Species micro-habitat use is gaining support as another important trait dictating trophic interactions [28, 35], and here we formulate terms which account for this trait in the ATN model. Differences in the use of micro-habitat (e.g., mainly foraging on the ground or in the vegetation) can mean that organisms rarely encounter each other and thus have a weaker than expected interaction.

The specific hypotheses we intend to test are: (i) In the absence of other species, consumption rates will be, to a large extent, determined by body size such that the impact of predators on prey, in simple two-species treatments, will increase with predator-prey body mass ratio up to the optimal body mass ratio. (ii) Habitat overlap between predator and prey affects predation rates such that predators which spend more time in the same habitat as their prey will have a stronger impact on prey populations. (iii) Presence of other species affects predator behaviour such that predators which are more likely to be intraguild prey to another predator, due to body mass ratio and shared habitat use, will spend more time avoiding the other predator and thus have a weaker impact on the shared prey in more complex (species-rich) treatments. (iv) A dynamical model parameterized using predator-prey body mass ratios and habitat overlap data will explain more (but not all) of the variation in species abundances than a model based only on body mass.

To maximize the amount of useful information collected relative to the amount of work required to collect it, we examine how the information content of the data that will be collected depends on the timing and frequency with which it is collected. Below we describe the original model presented in the literature as well as why and how we have altered it to describe missing traits and mechanisms we believe are important. We then describe how we design an experiment to test this model, including choice of predators and prey and traits to include. We finally use model sensitivity to parameter estimation to determine optimal timing, frequency, and subsampling techniques which allow us to best sample the experiment to obtain information necessary for model validation; this analysis is the main theme of our paper, as it is rarely used in ecological research.

## 1 Model: ATN with predator avoidance

We introduce a variation on the ATN model used in [8]. The original ATN model assumes Lotka-Volterra dynamics, with a functional response of type two which accounts for both intraspecific competition and predator satiation, and interaction parameters are generalized as functions of body mass. Here we introduce the parameter *ν*_*ij*_ to describe spatial overlap, with the hypothesis that species which spend more time in the same space will have a greater impact on each other. We assume that the only source of mortality for the duration of the experiment is predation.

Dynamics for the number of individuals *N*_*i*_ of species *i* are therefore given by

$$\begin{array}{c} \frac{dN_{i}}{dt}=r_{i}N_{i}-\sum_{j\in C_{i}}\frac{a_{ij}\nu_{ij}N_{i}N_{j}}{1+\sum_{k\in R_{j}}a_{kj}\nu_{kj}h_{kj}N_{k}+\sum_{l\in C_{j}}b_{0}a_{jl}\nu_{jl}N_{l}}, \end{array}$$ (1)

where *i*, *j*, *k*, and *l* are indices for species in the system. The first term in the differential equation (*r*_*i*_ *N*_*i*_) describes exponential growth of the population, with *r*_*i*_ the intrinsic growth rate of species *i* (which we assume to be zero-valued for predators for the duration of our experiment). The second term describes death by predation; we sum over all species *j* which consume species *i* ($j\in C_{i}$, for $C_{i}$ the set of predators for species *i*), and the fractions in this sum describe the population-level consumption of each species *j* on species *i*. The numerator is the possible attack rate of species *j* on species *i* and the denominator is a functional response that bounds this attack rate to physically reasonable levels. The first sum in the denominator is over all species *k* which are resources for species *j* ($k\in R_{j}$, for $R_{j}$ the set of prey for species *j*) and accounts for predator satiation. We introduce the second sum in the denominator, which accounts for intraguild interference. The parameters *a*_*ij*_ (attack rate), *h*_*ij*_ (handling time), and *ν*_*ij*_ (spatial overlap, or, similarity in habitat use) are parameterized by measurable traits, and definitions of these model parameters are summarized in Table 1.

**Table 1:** Definitions of model parameters. Left-aligned parameters (in shaded cells) are defined in terms of the corresponding right-aligned parameters (in unshaded cells). See (2) and (3) for details.

Parameter		Physical Meaning
*a*_*ij*_		Attack rate of species *j* on species *i*
	*a*_0_	Normalizing constant for encounter rates
	*R*_*opt*_	Optimal predator-prey body mass ratio
	*ϕ*	Sensitivity to predator-prey body mass ratio
	*W*_*i*_, *W*_*j*_	Body masses of species *i* and *j*
*ν*_*ij*_		Habitat use overlap for species *i* and *j*
	*v*_0_	Normalizing constant for habitat use
	*μ*_*i*_, *μ*_*j*_	Habitat uses for species *i* and *j*
*h*_*ij*_		Time for species *j* to handle *i*
	*h*_0_	Normalizing constant for handling time
	*W*_*i*_, *W*_*j*_	Body masses of species *i* and *j*
*b*_0_		Normalizing constant for predator interference

The quantity $\sum_{l\in C_{j}}b_{0}a_{jl}\nu_{jl}N_{l}$ describes the trade off that arises from some species balancing the competing requirements of being both predator and prey. In such cases, the time an organism spends avoiding potential predators will limit the time spent exploiting prey. We sum over the potential attack rates of all species *l* on a single individual of species *j* to account for time spent avoiding or evading species *l* while species *j* is attempting to capture its own prey. The parameter *b*_0_ is a scaling constant for the effect of this predator interference on the predation force of species *j*. For a group of cannibalistic predators, interference from one’s own species may not be distinguishable from general predator interference, and so we remove the intraspecific competition term *c*_*j*_(*N*_*j*_ − 1) as used in [8] from this version of the ATN model. We note that intraspecific interference due to potential predation is described by our formulation of the ATN model, but strictly competition-based intraspecific interference (as specified by a Beddington-DeAngelis functional response [36]) is not included.

As in [8], we assume that for species body masses *W*_*i*_ and *W*_*j*_ (corresponding to predator *i* and prey *j*), the allometric parameters are given by

$$\begin{array}{l} a_{ij}=a_{0}W_{i}^{1/4}W_{j}^{1/4}{\left(\frac{W_{j}/W_{i}}{R_{opt}}e^{1-\frac{W_{j}/W_{i}}{R_{opt}}}\right)}^{\phi },\\ h_{ij}=h_{0}W_{i}^{1/4}W_{j}^{-1/4}. \end{array}$$ (2)

We refer to [8] and references therein for a detailed defense of the allometric formulation for these parameters. To summarize, we rely on the assumptions that

1. the speed of an individual of species *i* scales with the quantity $W_{i}^{1/4}$,
2. there exists some predator-prey body mass ratio *R*_*opt*_ at which attacks by the predator on its prey are most successful, and
3. the time required for a predation event scales with decreasing predator-prey body mass ratios.

The quantity $a_{0}W_{i}^{1/4}W_{j}^{1/4}$ describes the rate at which individuals of species *i* and *j* encounter one another. The remainder of *a*_*ij*_ describes the likelihood of a successful attack for the encounter; the quantity inside parentheses is equal to 1 when the actual predator-prey body mass ratio (*W*_*j*_/*W*_*i*_) is equal to *R*_*opt*_ and decreases for *W*_*j*_/*W*_*i*_ ≠ *R*_*opt*_, while *ϕ* tunes the severity of this penalty. The handling time for an individual of species *j* to process an individual of species *i* decreases allometrically with the relative size difference between a predator and its prey (*R* = *W*_*j*_/*W*_*i*_) at a rate of *R*^−1/4^ (such that, relative to the size of a predator, smaller prey require a shorter handling time).

We introduce the parameter *ν*_*ij*_ to account for the effect of habitat use on encounter rates. For the set *Ω* of all modes of habitat use, we define the probability measure (*μ*_*i*_) such that evaluating $\mu_{i}\left(A\right)$ for some habitat A contained in *Ω* gives us the likelihood that species *i* occupies that habitat A. For example, one A in *Ω* might be “residing in foliage” and an entirely ground-dwelling predator would therefore have a measure $\mu_{i}\left(A\right)=0$. That is, *μ*_*i*_ is a mathematical way to describe observed species habitat use. For species pair *i* and *j*, we define the overlap parameter

$$\begin{array}{c} \nu_{ij}=1-v_{0}TV\left(\mu_{i},\mu_{j}\right)=1-v_{0}\underset{A\subset \Omega }{\sup}|\mu_{i}\left(A\right)-\mu_{j}\left(A\right)|, \end{array}$$ (3)

where *TV*(*μ*_*i*_, *μ*_*j*_) is the total variation distance [37] and 0 ≤ *v*_0_ ≤ 1 is a scaling factor for the importance of habitat use in determining spatial overlap. Total variation distance quantifies the difference between two measures, so *TV*(*μ*_*i*_, *μ*_*j*_) can be interpreted as the dissimilarity between the spatial distributions *μ*_*i*_ and *μ*_*j*_ of species *i* and *j*, and it takes on values between zero (no variation between *μ*_*i*_ and *μ*_*j*_) and one (maximal variation between *μ*_*i*_ and *μ*_*j*_). Continuing the example above, an entirely ground-dwelling predator and an entirely foliage-dwelling predator would have *TV*(*μ*_*i*_, *μ*_*j*_) = 1, since their spatial distributions are completely dissimilar. The corresponding overlap *ν*_*ij*_ = 1 − *v*_0_ is a quantification of the similarity of habitat use for the two species in our example, with *v*_0_ indicating how much weight we give to the assumed distributions *μ*_*i*_ and *μ*_*j*_. If *v*_0_ = 1, then we give full consideration to the assumed distributions (*ν*_*ij*_ = 0 and the predators do not overlap in our model). If *v*_0_ = 0, we ignore the assumed distributions (*ν*_*ij*_ = 1 and the predators completely overlap in our model). For a more detailed explanation of this formulation with examples from our experiment, see S1 Appendix.

Our primary goal in choosing traits to model is that they significantly affect population dynamics. However, we must weigh this goal against the model’s general relevance for differing communities and environments outside the scope of our experiment, where we eventually hope to evaluate its performance [38]. We must also consider the tractability of our model; a model with too many parameters to feasibly identify or which fluctuates wildly for relatively small changes in model parameters cannot reliably be used to investigate questions about physical processes. Although our formulation of overlap is not driven by physical mechanisms, it is amenable to inclusion in the ATN model, which assumes encounters rates increase with mutual distance covered. Inclusion of *ν*_*ij*_ scales species encounter rates so that they increase with mutual distance covered *in the same habitat zones*. Additionally, this formulation is flexible and can be adapted to any study system; we can define the set *Ω* as is most-relevant for the expected species behaviors and measure *μ*_*i*_ in any new habitats.

## 2 Designing the experiment

### 2.1 Choosing the study system

To test this model requires a food web which is easily manipulated in the lab, where generation time is sufficiently brief to observe population dynamics over a short time period, and where predators and prey vary in the traits of interest, body mass and habitat overlap. By selecting both predators and prey which vary in these traits, we can additionally investigate the importance of a match between predator and prey traits.

**Fig 1:**
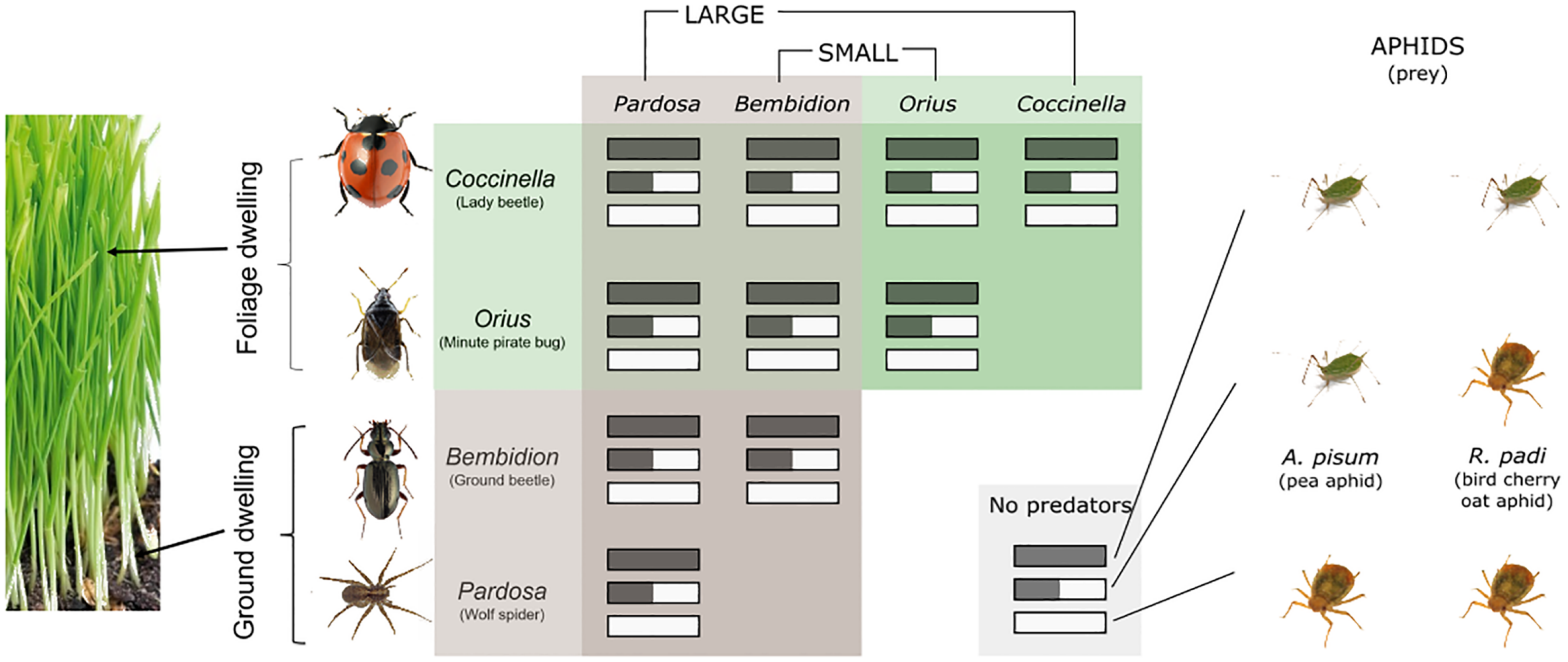
An overview of the experimental design and the hypotheses we test. We select two predators which reside primarily in the foliage and two on the ground. Of those two, one is large and one is small such that each combination of size and habitat use is covered. Each predator is combined with each other predator, including itself, to cover all one- and two-predator combinations. We also select two species of prey, and within each predator combination, each prey combination is represented (shown by horizontal bars of one or two colors). Thus, including controls containing no predators, we have 33 predator-prey combinations.

We use modules from a simplified agricultural food web to test the model, which we manipulate in a replicated cage experiment in greenhouses (Fig 1). In addition to the reasons listed above, we choose this foodweb for its important applied relevance—crop pest control using natural predators—and because we have data from a previous experiment to use as a basis for the predictions outlined here. Furthermore, the prey items (aphids) reproduce rapidly, allowing us to determine the effect of predators on their population growth in a tractable period of time. For this reason, we limit the length of the experiment to 8 days so that aphid growth is neither limited by quality of remaining resources nor complicated by the production of alates, although these mechanisms are important in describing long-term population dynamics. We focus our efforts on modelling the effect of predator interactions on aphids in the absence of processes which introduce uncertainty to our model, and we monitor for the appearance of alates during the experiment to ensure that these assumptions are met.

To determine the effect of body size and habitat overlap, we select predators varying in both of these traits. We choose two predators which are primarily ground predators (Wolf spiders of the genus *Pardosa* and ground beetles of the genus *Bembidion*) and two which primarily reside in the foliage (lady beetle *Coccinella septempunctata* and the minute pirate bug *Orius majusculus*). While these predators primarily reside either in the foliage or on the ground, there is still substantial overlap and all predators can encounter one another. We note in preliminary trials that *Bembidion* is the most restricted in habitat use, while *Pardosa* sometimes climb the plants, and both of the foliage predators spend time on the ground. We choose phylogenetically diverse predators to determine whether the importance of body size and habitat use are valid despite phylogenetic differences. *Pardosa*, *Coccinella*, and *Bembidion* are collected in the field at locations 59.740 °N, 17.680 °E and 60.047 °N, 17.981 °E, in areas surrounding Uppsala, Sweden. *Orius majusculus* is purchased from Lindesro AB, Helsingborg, Sweden.

We select a large and a small predator for both foliage and ground predators to determine the effect of body size (Fig 1). To also compare the importance of a match between predator and prey traits, we use two prey species (both aphids) differing in body size and habitat use. The larger aphid (*Acyrthosiphon pisum*) lives primarily on the underside of bean leaves, while the smaller aphid (*Rhopalosiphum padi*) grows on barley plants and resides low down on the stem where it is available to ground-dwelling predators. The choice of these two prey species necessitates the choice of barley and beans as plants, which we sow in five alternating rows (three rows of barley, and two rows of beans). Beans are planted first, as they take longest to grow. We plant barley four days after beans, and seven days after that we uniformly thin the seedlings (leaving 15 tillers in every barley row and 10 tillers in every bean row) before introducing the aphid population. After two days, we introduce the predator population and monitor aphid abundances for the following eight days. We state the initial abundances of all species, as well as their body masses, in Table 2.

**Table 2:** The body masses and initial abundances for species in our study system. We note that the initial abundance for a single-species treatment is doubled, as if we were introducing two populations of the same species.

	*A*. *pisum*	*R*. *padi*	*Bembidion*	*Coccinella*	*Orius*	*Pardosa*
mass (mg)	0.6706	0.1550	2.145	37.4636	0.58	17.72
initial density	75	75	20	2	20	10

Having a range of predator body sizes also allows us to explore intraguild predation and predator avoidance as a result of body mass and habitat overlap. We hypothesize that the closer one predator is to the optimal predator-prey body mass ratio of a larger predator, the more likely that the smaller predator will be the victim of intraguild predation and thus spend time avoiding the larger predator instead of consuming aphids. Similarly, we hypothesize that with greater overlap in habitat use, smaller predators are more likely to become intraguild prey and thus spend time avoiding the intraguild predator. To test these hypotheses, we test all combinations of two predators with each of the prey species alone and with both prey species; this allows us to explore how a predator’s traits match with different prey as well as how the effect of predators on each other may change their impact on the prey. Each combination of predators and prey is replicated six times in caged mesocosms in the greenhouse, with the number of replicates selected as a result of a previous experiment. Including controls for each of the prey treatments, this yields 33 different predator-prey combinations (Fig 1). We will use the control treatments to determine the intrinsic growth rate of the aphid populations, after which we will rely on the predator-treated mesocosms to specify the remaining model parameters.

### 2.2 Determining parameter sensitivities and critical sampling times

A standard approach when designing ecological experiments tends to be to distribute data collection at regular intervals without much consideration of the information content inherent to various sampling dates. To effectively estimate parameters for a dynamic model, we must collect sufficient data throughout an experiment, but obtaining data at an appropriate temporal scale is a time-consuming and expensive endeavour. It is therefore advantageous to know the optimal timing for data collection during the experiment, with the goal of using these data to estimate model parameters *a*_0_, *R*_*opt*_, *ϕ*, *h*_0_, *b*_0_, and *v*_0_ (from which the higher-level parameters *a*_*ij*_, *h*_*ij*_, and *ν*_*ij*_ can be computed as given in (2)). We will estimate model parameters using an “inverse problem” methodology, in which model dynamics are fit to time-series data using some predetermined cost function of the collected data. Data from different days can vary in how much information they provide for the solution of the inverse problem, and we want to focus our effort on those days which provide the most information. To ensure that maximal information is present in the collected data, we examine the sensitivity of model output (population densities) to parameter inputs as time progresses. We refer to sensitivities of great magnitude (regardless of sign) as “high”, indicating that model solutions depend strongly on a given parameter. To effectively estimate parameters for our model, we must collect data on days with high sensitivity; we obtain less information about the parameters by sampling on days with low sensitivity. We are primarily concerned with the sensitivities of aphid population densities with respect to model parameters, since we cannot obtain predator population data during the experiment.

We compute the sensitivity of a population density *N*_*i*_ with respect to a parameter *θ*, $s_{i}^{\theta }=\frac{dN_{i}}{d\theta }$, by solving the sensitivity equations [39, 40] which are given in S2 Appendix. To facilitate comparison of sensitivities between treatments, we compute the relative sensitivity (similar to elasticity) [41, 42]$sr_{i}^{\theta }\left(t\right)$,

$$\begin{array}{c} sr_{i}^{\theta }\left(t\right)=\frac{\theta }{N_{i}\left(t,\theta \right)}s_{i}^{\theta }\left(t\right), \end{array}$$ (4)

and to avoid giving undue importance to aphid populations approaching zero, we do not normalize by the population when *N*_*i*_(*t*, *θ*) < 1. We present sensitivity results for *θ* = *a*_0_, *ϕ*, *v*_0_, *h*_0_, *b*_0_ using parameter values *a*_0_ = 24 × .9, *ϕ* = 1, *v*_0_ = 1, *h*_0_ = 2/24, *b*_0_ = *h*_0_, *R*_*opt*_ = 60 for *Bembidion*, *R*_*opt*_ = 115 for *Coccinella*, *R*_*opt*_ = 1 for *Orius*, and *R*_*opt*_ = 60 for *Pardosa*. We cannot know the model parameters prior to running the experiment, and so our scaling parameters are informed by estimates from similar mesocosm experiments [24] and *R*_*opt*_ is estimated from personal observations of predators during planning stages of the experiment. We choose these parameter values because they are, to the best of our knowledge, physically reasonable. We stress that these “preliminary” values will likely differ from actual estimates in our experiment. However, if there is not a dramatic difference between these values, preliminary approximations are useful for suggesting sampling times (as utilized below). We additionally computed model sensitivities for a range of nearby parameter values and obtained similar results.

**Fig 2:**
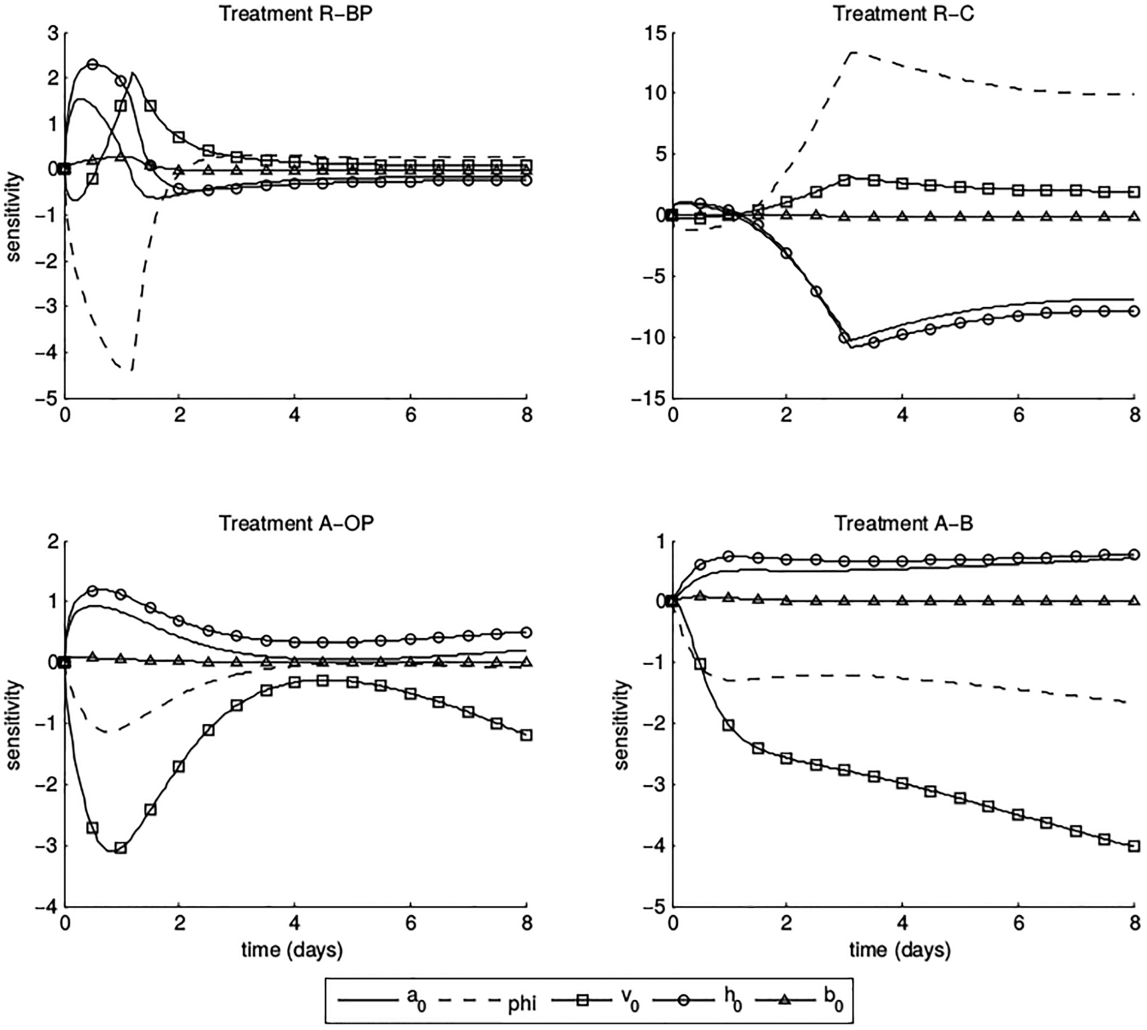
Sensitivities of aphid population abundances with respect to model parameters. Upper left: *R*. *padi* with predator treatment *Bembidion-Pardosa*. Upper right: *R*. *padi* with predator treatment *Coccinella*. Lower left: *A*. *pisum* with predator treatment *Orius-Pardosa*. Lower right: *A*. *pisum* with predator treatment *Bembidion*. Sensitivities are plotted in time and different parameters are indicated by line style.

From control (no predators) cage “trial runs” during planning, we estimate intrinsic growth rates *r* = 0.3007 for *A*. *pisum* alone, *r* = 0.3211 for *R*. *padi*, and *r* = 0.2453, *r* = 0.2591 for *A*. *pisum* and *R*. *padi* (respectively) in a combined treatment. We plot in Fig 2 the sensitivities of aphid populations to model parameters for four cage treatments (described in Table 3), which exhibit the types of behavior we see in the sensitivities as a function of time. We note that we do not consider sensitivity results for growth rates *r*, as we know that sampling at the beginning and end of the experiment is sufficient for the estimation of an exponential growth rate. We additionally do not include sensitivity of model solutions to *R*_*opt*_ in our consideration of sampling protocol. We manually specify values of *R*_*opt*_ which describe an impact on the aphid population; without corroborating this decision with estimates using experimental data, we are not confident in the resulting reports of parameter sensitivity.

**Table 3:** Key for cage treatments referenced in Fig 2.

Treatment	Prey	Predator 1	Predator 2
R-BP	*R*. *padi*	*Bembidion*	*Pardosa*
R-C	*R*. *padi*	*Coccinella*	*Coccinella*
A-OP	*A*. *pisum*	*Orius*	*Pardosa*
A-B	*A*. *pisum*	*Bembidion*	*Bembidion*

The first type of sensitivity behavior (represented by treatment R-BP) is the most common and characterized by high sensitivities on or before *t* = 2, which quickly decrease to low levels for the remainder of the experiment. Cages of this type correspond to aphid populations which the model predicts will be quickly decimated by the predator treatment and do not recover. The second type (represented by treatment R-C) is characterized by low sensitivities until *t* = 2, after which sensitivities quickly increase to their peak around *t* = 3 and remain at high levels for the duration of the experiment. In these cages, the modeled aphid population does experience eventual decimation, but the decline is sometimes slightly delayed. The third type (represented by treatment A-OP) exhibits high sensitivity early in the experiment, which decreases to low values around *t* = 4 and then increases slightly towards the end of the experiment. The modeled aphid population under these treatments experiences a drastic decline at the beginning of the experiment but manages a slight rebound when predators become scarce later in the experiment. The final type (represented by treatment A-B) has constant or increasing sensitivities for most of the experiment, with sensitivities increasing the most before *t* = 2. Treatments of this type do not result in a drastic decline in the modeled aphid population; the population is increasing for at least several days of the experiment.

We note that our sensitivity results are local to the assumed parameters in model formulation and therefore may change for different parameter inputs. That is, if any parameters estimated from experimental data are significantly different from the values we assume above, the model sensitivities to *all* parameters may differ from what we present here. To minimize this risk, we obtain assumed parameter ranges using estimates from a related experiment and verify that similar peaks in sensitivity are obtained for different parameter values in this range. We cannot draw conclusions about the specific cages which belong to each of these categories for the experiment, where true model parameters may be significantly different from what is assumed here. Rather, we present an example of the types of behaviors that model sensitivities might exhibit, regardless of the treatments under which they truly occur. Based on these results, sampling on days *t* = 2, 4, and 8 will yield data with high information content related to all model parameters, in at least some treatments. In the interest of obtaining maximal information in treatments with rapid aphid population decline, we suggest additional samples on days *t* = 1 and 3. We obtain less information with repeated sampling later in the experiment, when the population has already been decimated; the rate of decimation is of greater interest. Under this sampling scheme, we can be as confident as possible that the collected data contain sufficient information for parameter estimation.

**Fig 3:**
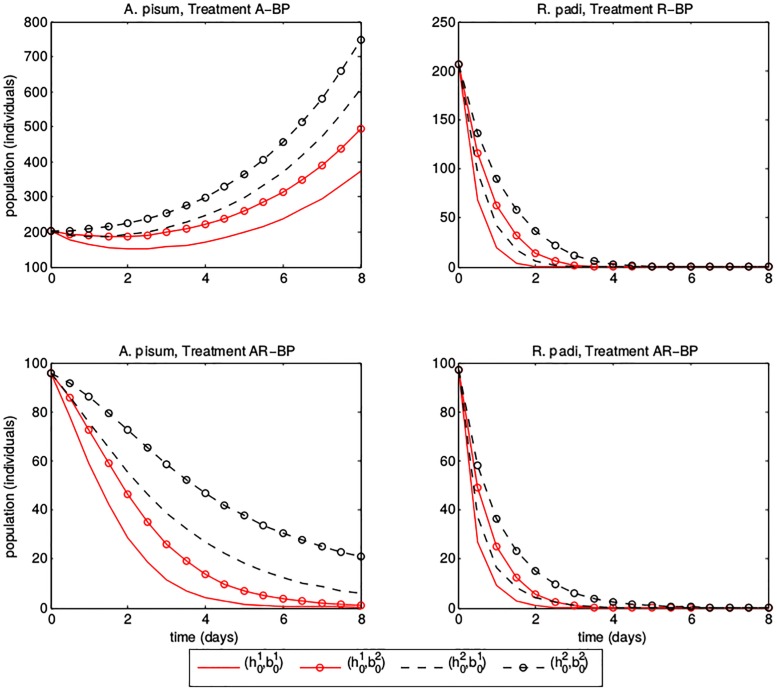
Model trajectories for aphid populations under predator treatment Bembidion-Pardosa. Upper left: *A*. *pisum* aphid treatment. Upper right: *R*. *padi* aphid treatment. Lower left: *A*. *pisum* in combined aphid treatment. Lower Right: *R*. *padi* in combined aphid treatment. We plot model solutions for all combinations of parameters $h_{0}^{1}=2/24$, $h_{0}^{2}=3/24$, $b_{0}^{1}=2/24$, and $b_{0}^{2}=9/24$.

However, use of the above sampling scheme does not immediately imply that model parameters are realistically identifiable. When solving the inverse problem, we may find that some parameters are difficult to identify simultaneously; despite describing different mechanisms, these parameters might have similar effects on prey dynamics and therefore be indistinguishable when validating the model against experimental data. To illustrate this, we present a contrived example of a potential identifiability issue in Fig 3, where we consider model solutions for a single predator treatment (*Bembidion-Pardosa*) using different parameter values. We choose two reasonable values of *h*_0_ based on the results from [24] ($h_{0}^{1}=2/24,h_{0}^{2}=3/24$). Since we introduced *b*_0_ to the model, we do not have prior results to consult; we consider $b_{0}^{1}=2/24$ and $b_{0}^{2}=9/24$ to capture a broad range of resulting model dynamics. Suppose we were trying to determine the value of *h*_0_ from population trajectories; if $b_{0}=b_{0}^{2}$, we see a significant difference in the *A*. *pisum* population on day 8. If populations were close to 450 under treatment A-BP and close to 0 under treatment AR-BP, we could conclude that $b_{0}=b_{0}^{2}$ and $h_{0}=h_{0}^{1}$. However, suppose we did not know that $b_{0}=b_{0}^{2}$ and instead assumed that $b_{0}=b_{0}^{1}$. Since error in the data is as likely to over- as under-estimate the aphid population, we might erroneously conclude that $h_{0}=h_{0}^{2}$ (dashed line, no marker) instead of $h_{0}=h_{0}^{1}$ (solid line, no marker) based on a noisy observation of the true solution (solid line, circle marker). Given the noise inherent to experimental data (in particular, noise which will increase with the size of the population we sample), it is unlikely that we could distinguish between these two population trajectories. This problem is inherent to the model and we cannot necessarily anticipate where it may arise prior to parameter estimation. However, we can guard against this problem by sampling sufficiently often in time to outweigh experimental noise. If such sampling is not feasible, comparing parameter results across different treatment types can improve identifiability, as we expect these problems to vary across treatments.

### 2.3 Determining feasible sampling strategies for parameter estimation

In defining the sampling protocol, we must balance what is a reasonable undertaking with the data required for parameter estimation. Since different methods of data analysis have different criteria for meaningful information content, we must identify the method of analysis when establishing sampling protocol. This choice determines the frequency with which we must collect data and the sampling strategies we can implement. We employ a data-driven least squares minimization to estimate model parameters, as described in [39, 40]. We note that other methods exist for the estimation of model parameters (see, for example, examining terminal interaction strengths as in [8]). Solving a least squares inverse problem allows us to fit model dynamics to data in time, preserving maximal information about physical processes. However, convergence of this solution requires substantial, temporal data, and we must therefore utilize increased sampling rates.

Obtaining these data is a time-consuming and expensive process and, with limited resources, becomes a trade-off between sampling thoroughly at a single time or sampling frequently in time. There is no consistent translation of this requirement to some amount of data required to obtain a sensible solution to the inverse problem. However, we tentatively assume that to estimate our five model parameters, we should inform the inverse problem with at least ten data points. Grouping our cages into cohorts of the three aphid treatments, with predator treatments fixed, yields nine data points when using our proposed samples on days *t* = 2, 4, 8. The alternative of grouping our cages into cohorts of the ten predator treatments, with aphid treatments fixed, for 30 data points is perhaps drastic (especially since we can only theorize that dynamics will be the same for all predators, despite phylogenetic differences). We therefore compromise by adding an additional sample on day *t* = 6, which may not be a day with particularly high parameter sensitivities but does give additional information. This choice still presents an enormous effort, requiring many man-hours of lab work. We therefore consider the potential for subsampling of the aphid population in order to obtain data more quickly (with less effort) on these days; due to resource availability, it is crucial that we establish a subsampling protocol for the experiment.

To subsample the aphid population, *N*_*j*_, at time *t*_*j*_ in a cage with *T* tillers (individual plants on which aphid populations are counted), we count the number of aphids on *n* < *T* tillers, ${\tilde{N}}_{j}^{n}$, and scale by *T*/*n* to obtain an estimate of the aphid population, $N_{j}^{n}=\frac{T}{n}{\tilde{N}}_{j}^{n}$. We investigate the effect that any error in $N_{j}^{n}$ might have on our ability to estimate model parameters. We first consider this problem in predator-free control cages for *R*. *padi*, for which we had previous data to consult. In these cages, we assume strictly exponential growth of the aphids at some rate *r*_0_, and we estimate the average growth rate $\bar{r}$ in six replicate cages to use as an approximation to *r*_0_ in the ATN model. From the per-tiller counts in these six cages, we construct synthetic data for aphids with growth rate $\bar{r}$ (see S3 Appendix for details). We consider subsampling strategies in which *n* = 5, 6, …, 90 tillers are counted and estimate the growth rate *r*^*n*^ from the approximated population $N_{j}^{n}$. We compute the resulting error $\left|r^{n}-\bar{r}\right|$ for all *n*, and repeat this process for 200 synthetic data sets.

**Fig 4:**
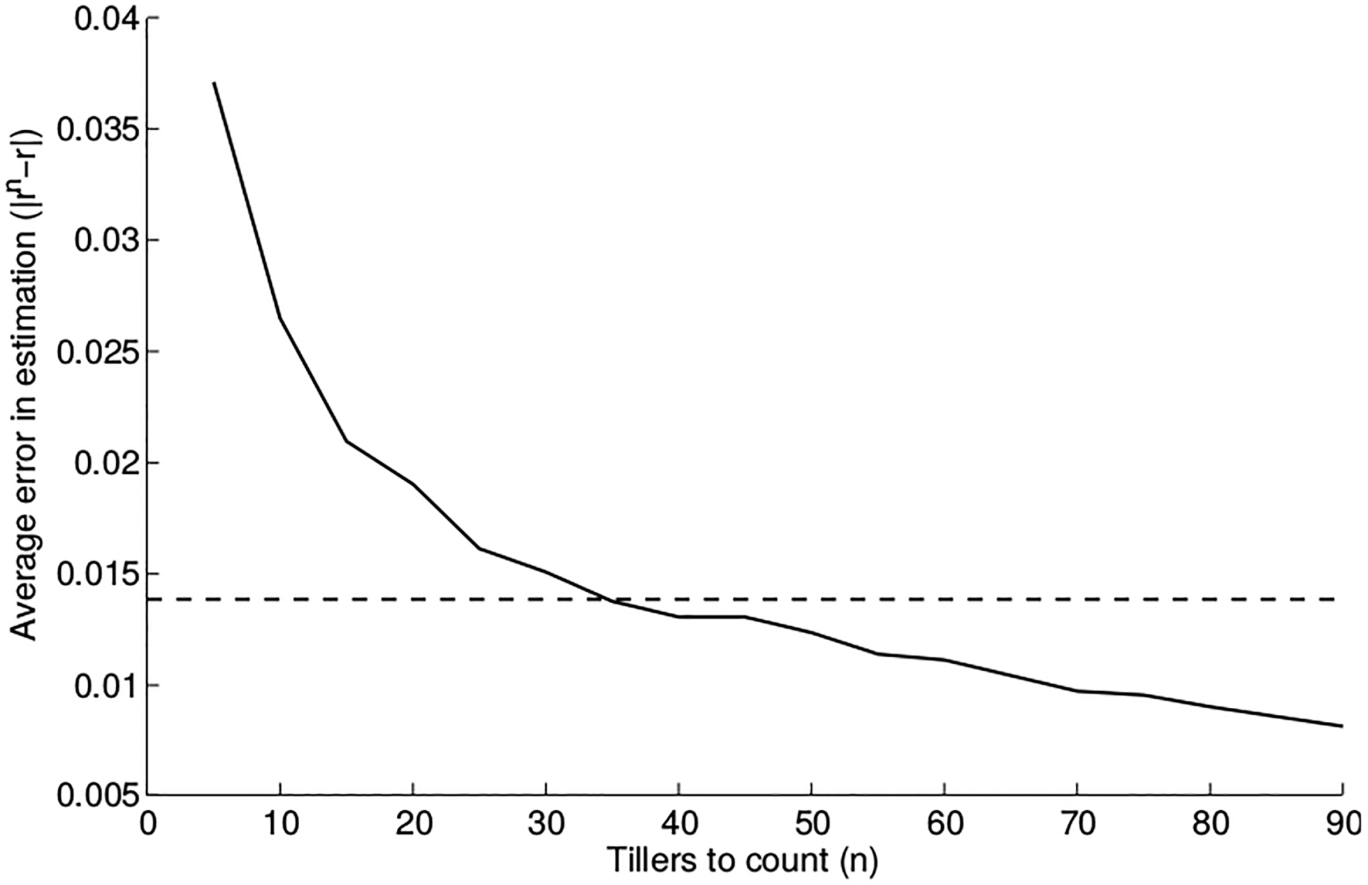
The average value of $\left|r^{n}-\bar{r}\right|$ across 200 synthetic cages. The horizontal dashed line indicates the average value of $\left|r-\bar{r}\right|$ for parameter estimates using the true data set.

We plot the average error $\left|r^{n}-\bar{r}\right|$ for each subsampling strategy in Fig 4. The dashed line in the figure indicates the average value of $\left|r-\bar{r}\right|$ for the estimated growth rates in the six true data sets. We note that although the strategy prone to the least error is to sample all 90 tillers, the accuracy gained from sampling additional tillers drops significantly around *n* = 25 tillers. Sampling around *n* = 40 tillers results in error that is on the same scale as the average value of $\left|r-\bar{r}\right|$. That is, the error induced by subsampling is no more drastic than the natural variation we see in parameter estimates across controlled replicates; if we are comfortable averaging out the latter and incorporating this parameter in our model, then the former should be similarly manageable. We therefore conclude that sampling 45 out of 90 tillers (or, 1-in-2 subsampling) yields sufficiently accurate parameter estimates. If the burden of data collection is too high, sampling 30 out of 90 tillers (1-in-3 subsampling) is justifiable, and sampling as high as 60 out of 90 tillers (2-in-3 subsampling) seems to be a very safe, if intensive, strategy.

**Fig 5:**
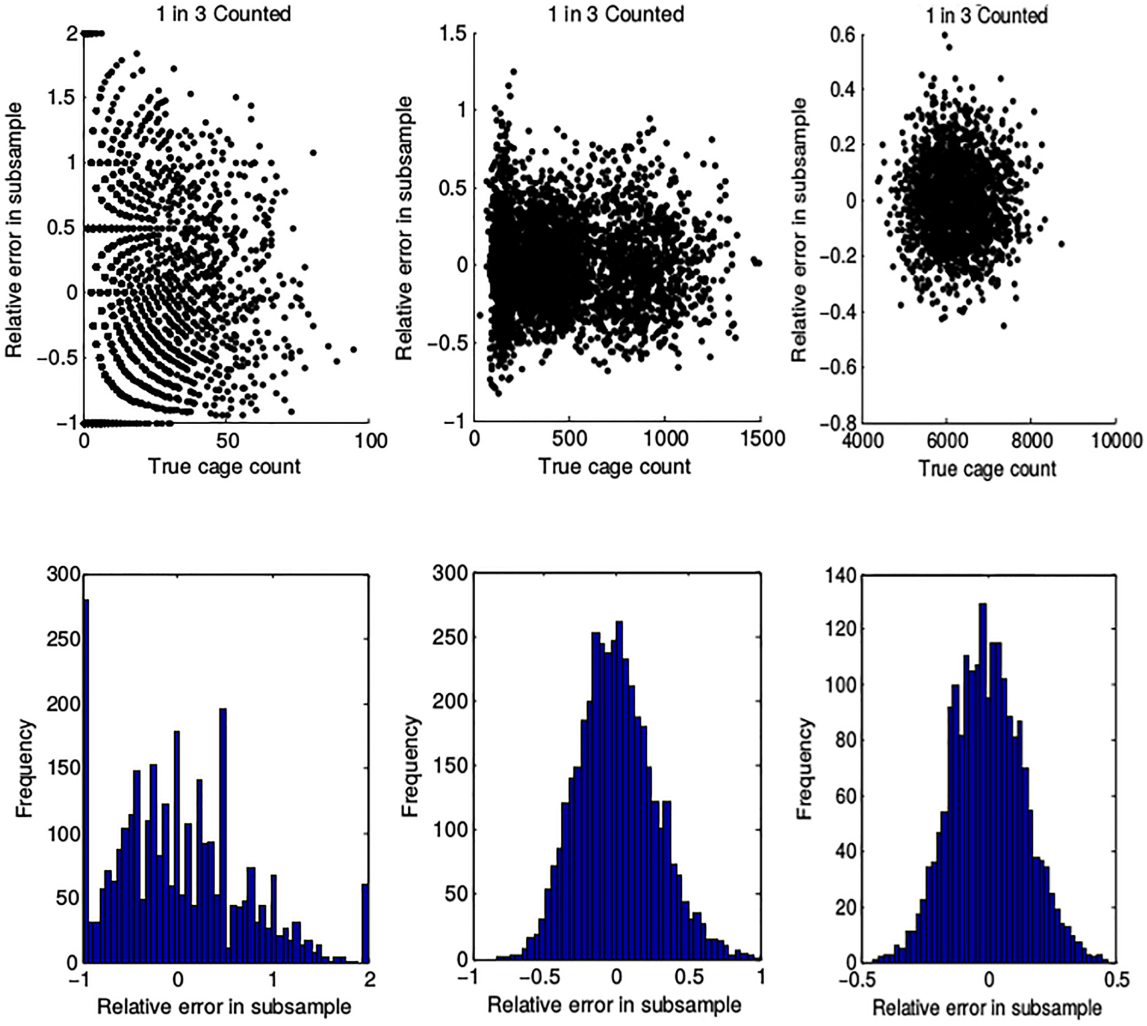
Normalized errors induced by subsampling at a 1-in-3 rate for synthetic data. Left: data in the ranges of [0, 100]. Middle: data in the ranges of [150, 1500]. Right: data in the ranges of [4000, 9000].

We next consider the effect of error induced by subsampling on our estimates of ATN model parameters. We obtained per-tiller aphid counts for experimental cages with *R*. *padi* as the basal species, but sometimes under different predator treatments than our intended study communities [24]. Additionally, previous data are not sampled sufficiently in time to permit the estimation of ATN model parameters. We must directly infer from these experimental data the distribution of noise induced by subsampling (see S3 Appendix for details) and add this noise to a simulation of cages which we assume perfectly follow ATN dynamics. We present in Fig 5 the scatter plots and histograms of the normalized error in aphid population counts induced by subsampling synthetic data for three categories of aphid densities, corresponding to full-cage counts in the ranges of [0, 100], [150, 1500], and [4000, 9000]. We only present results for the 1-in-3 subsampling rate here; at higher subsampling rates, the behavior of the error is similar.

We conclude that for full-cage populations greater than 150 aphids, the normalized error induced by subsampling *n* tillers is normally distributed, with variance decreasing as the aphid population increases. However, we note that when the aphid population is at very low values, it is easier to make the worst possible over- and under-estimates of the population when subsampling. There is therefore a clear bias towards the upper and lower limits of the error induced by subsampling in this range. When generating synthetic data with errors sampled from the distributions in Fig 5 and dynamics given by ATN model solutions for use in an inverse problem, visual fits to the true model solution are poor for aphid populations below 150 (results not shown). Although the error in our parameter estimation did not vary considerably in these cases, we caution against implementing a subsampling strategy for cages with low population densities until more information is available.

An additional challenge in employing this method of parameter estimation is that the accuracy of results is dependent on the study system’s adherence to both the *mathematical model of dynamics* and the *statistical model of error* in the data. In particular, misspecification of the statistical model can result in inverse problem solutions which appear to fit the data without matching underlying behavior. This is a problematic outcome, as it is impossible to tell from standard errors in our estimates and visual fit alone if the data are accurately described by the models [40]; model-based analysis in this situation could be wrong without any indication of the underlying issue. To avoid this, the statistical model is often revised iteratively, with methods which either include an assumption of the underlying dynamics or which rely entirely on information present in the data, once experimental data are available (see [43, 44] for implementation of these methods). When establishing the experimental sampling protocol, we must consider the underlying statistical model of error that such a protocol suggests and how we might be able to identify this model.

A previous cage experiment [24], which we have used as the basis for this work, employed destructive sampling on the final day of data collection (to obtain estimates of aphid and predator abundances) and only collected aphid abundance data on one additional day during the experiment. When a cage is destructively sampled, each plant is cut at ground level and removed from the cage before aphids are counted. Aphids that are obscured from view within the plants can be found with destructive sampling, and we therefore expect that destructive sampling yields a more accurate count of the population. However, it is not possible to destructively sample a cage more than once, meaning we cannot sample this way before the end of the experiment. Unfortunately, this means we cannot describe the relationship between the error in non-destructive and destructive samples at all aphid density levels without destructively sampling over a range of predator treatments for the duration of the experiment. Without the space and resources to destroy a set of cages on every day that data are collected, we refrain from destructively sampling the aphid population on the final day. We are therefore able to statistically model error with fewer unquantifiable assumptions, despite trading away the added accuracy of a destructive count on a single day.

### 2.4 Fitting and evaluating the model

Once the experiment is performed, we will parameterize our formulation of the ATN model using the experimental abundance data in a least squares inverse problem (as summarized at the beginnings of Sections 2.2 and 2.3, and detailed in [39, 40]). We will quantify statistical properties of noise in our data, which permits the formulation of a weighted cost criterion describing the difference between model predictions and empirical observations. By minimizing this cost, we obtain a best-fitting set of model parameters. We will first establish a baseline for aphid growth using data from the control treatments, after which we will estimate ATN model parameters using data from predator-treated mesocosms. By fitting the same model parameters to treatments which utilize a phylogenetically diverse group of predators, we test the generalizability of the trait-based model.

In order to compare the importance of habitat use and predator interference (as described in our model by *ν*_*ij*_ and *b*_0_, respectively), we will repeat this fitting for three models

1. the full model, as described in section 1,
2. a partially-reduced model, where *b*_0_ = 0, and
3. a fully-reduced model, where *b*_0_, *v*_0_ = 0.

The performance of each model will be compared based on

(i) the fit of the model, as quantified by the cost criterion described above,
(ii) precision (i.e. standard errors) of parameter estimates,
(iii) realism of estimated parameter values, and
(iv) realism of associated processes (e.g. feeding rates).

In comparing performance by (i), a lower cost indicates a better model fit; however, we formalize this by using a statistical comparison test for nested models, which corrects for the number of parameters in each model. To evaluate (ii), we seek smaller standard errors associated with our estimates; a small standard error indicates high confidence in the estimate, which requires that the model is sensitive to the parameter while reasonably fitting true dynamics. Evaluation of (iii) and (iv) requires that we consult literature or supplemental empirical testing; the realism of parameter values and processes allows us to assess how accurate the models are.

## 3 Discussion

We have outlined a theory to test, developed a model which distills that theory into a mathematical relationship, and designed experimental protocol to test that model, including the optimal sampling method to minimize potential difficulties in estimating model parameters. Taxa and treatments are chosen to address our trait-based hypotheses within the framework of our ATN model, and combined in the design laid out in Fig 1. In order to attain sufficient information for model validation, we sample on days 2, 4, 6, and 8. If we are constrained by resources and cannot sample at this rate, we expect that aphid populations will be most sensitive to model parameters on days 2, 4 and 8. For treatments under which the aphid population is rapidly decimated, it may be necessary to sample more frequently at the start of the experiment. In these cases, we may also sample on days 1 and 3. From the available information, we conclude that population data obtained by subsampling 2-in-3 or 1-in-3 tillers would be sufficiently accurate under most treatments. However, since this analysis is based on data from an experiment with different prey treatments than we use, we fully sample whenever possible.

We note that our proposed sampling schemes are intrinsically linked to the assumed ATN model for species behavior and statistical properties of the data we intend to collect. Because of this, we cannot make general conclusions about the amount of data necessary for validation of a given model, or the frequency with which such data should be collected (except for models and study systems sufficiently similar to our own). Instead, we make the case that sampling schemes should optimize the expected information content of the resulting data set, in the context of the proposed model for system dynamics and anticipated experimental noise. By designing the experiment explicitly to test the model, we substantially increase our confidence that any variation the model does not capture is due to mechanisms we have missed or misrepresented in the model, as opposed to simply insufficient data or data collected at incorrect temporal resolutions. This can then form the basis for future studies investigating additional traits or mechanisms in the iterative process of refining both the model and experiments testing it [2]. By explicitly presenting our route through this process, we prevent the possibility of short-circuiting it by retrospectively creating hypotheses to explain our data [45].

We first explicitly link the experiment with the model, allowing a clear test of how the traits we seek to study affect food web dynamics. However, it is equally valuable to work in the opposite direction, formulating our model with the knowledge that we will be fitting it to an unrealistic mesocosm experiment. By measuring habitat use throughout the experiment and including it in our formulation of overlap, our model implicitly addresses the fact that species may be forced by the experimental design to share habitat. Our model therefore allows for results to be generalized to communities where species habitat use might be differently defined, which is amenable to our eventual goal of extending an experimentally validated model to naturally occurring communities.

In specifically formulating our model to address a trait-based hypothesis, we guarantee that each parameter’s effect on model output is an expression of the importance of an ecological mechanism we seek to study. Solutions to time-varying sensitivity equations therefore reveal intervals over which population data are most-influenced by the mechanisms we have designed this experiment to study, and temporal sampling protocol must be informed by these results. Failure to obtain data on days with high sensitivities might lead to the conclusion that an experiment was not sufficiently thorough to determine the importance of a particular mechanism or, even more alarmingly, that the mechanism does not significantly affect dynamics. For our design, we note that sensitivity peaks across different two-day intervals for a variety of experimental treatments and identify treatments where it might be necessary to sample more frequently at the start of the experiment. By identifying critical sampling times for maximal information prior to running the experiment, we ultimately save time and resources and, most importantly, safeguard against the possibility of collecting data which are useless in model validation [46].

It is an unfortunate reality that unforeseeable circumstances will sometimes result in missing data where experimental results cannot be obtained, making model validation difficult. In our experiment, we cannot guarantee that there will be sufficient time in the day to obtain the necessary data at the experiment’s peak, when populations have reached maximal values. In addition to identifying a temporal sampling protocol, it is therefore equally important that we determine a viable subsampling protocol before starting the experiment. We conclude that once the population reaches a threshold value, subsampling is a viable option. At low population densities, subsampled populations might poorly estimate the true population size, and so we prioritize fully sampling these populations when there is insufficient time for the full sampling of all populations. Moreover, our quantification of the error induced by subsampling will be necessary in informing any uncertainty quantification for our estimated parameters, if subsampling is implemented. In adapting this hybrid subsampling protocol, we guarantee that the best possible data are collected whenever possible. In the event that we cannot fully sample, we collect imperfect population data with quantifiable error for all treatments; from a mathematical perspective, this is preferable to the alternative of collecting perfect data for a handful of treatments and obtaining no information on the remaining treatments.

The final step is to implement this experiment and use the data to estimate model parameters that yield a best-fitting solution to observed dynamics, which we will present in future work. However, in anticipation of this goal, we explore potential identifiability issues which, as with many ecological models, exist in our formulation of the ATN model. Although we have designed the experiment to maximize information content in the data, we demonstrate that it is necessary to investigate possible non-unique parameter solutions when fitting the model to these data and potentially augment our parameter estimation with some empirically measured parameters. The intent in fitting a model to data from an ecological community is not only to numerically identify a specific parameter, since the parameter value may not be generalizable to other communities. Instead, our goal must be to describe the important mechanisms driving dynamics. If different parameter sets can fit the data equally well but tell different stories about necessary traits in describing trophic interactions, then a failure to recognize these identifiability issues renders conclusions about underlying behaviors questionable.

A challenge in model validation is the ease with which confounding factors can derail our understanding of the conditions for which a particular model is appropriate. In designing our experiment, we must balance the need for controlled conditions with generalizability. We utilize predators of diverse phylogeny in order to test the strength of these traits in determining trophic interactions *despite* phylogenetic differences. However, we make a number of simplifying assumptions and omissions which would otherwise be valuable additions to the model. We design our experiment to control for predator population growth and complicated modes of aphid reproduction, limiting our ability to test long-term model applicability. Moreover, a multitude of other traits affect trophic interactions, and with the exception of predator avoidance, we do not consider behavioral additions to the model. In particular, predator avoidance by prey is an important aspect of population viability which we exclude in this experiment. As a preliminary attempt at advancing a trait-based model, we choose two traits which we believe to be particularly important and attempt to precisely quantify the effect of these traits on aphid dynamics and intraguild predation. By omitting additional mechanisms, we ensure that the experiment is specific to our traits of interest and maximize our confidence that the effects of these traits will be captured by the empirical data and model fitting. After establishing a baseline model for these simple interactions, we must formulate and test new terms which are appropriate under complicated conditions.

The future of predictive ecology lies in the successful implementation of models to predict changes in natural communities. In order for these models to produce credible results, they must be based on and tested by empirical experiments. Moreover, these experiments must be optimally designed such that sufficient data can be extracted to inform the model [47]. By presenting this process before conducting the experiment, we have been forced, a priori, to explicitly establish our hypotheses and the rationale behind our experimental design [2, 45, 48]. This ensures that we have designed the experiment in a way which maximizes its relevance to the model. When validating the model with experimental data, we will be able to investigate the importance of modeled traits with maximal confidence and, if necessary, conclude that additional traits must be investigated to sufficiently describe observed behavior. The results of the experiment will inform our efforts to build an appropriate model for these dynamics which can be applied to different study systems, furthering our ability to describe and predict behaviors in natural ecosystems.

## Supporting information

S1 Appendix Formulation of habitat use. (PDF)

S2 Appendix ATN sensitivity equations. (PDF)

S3 Appendix Expanded subsampling results. (PDF)

S1 FigFit of exponential model to control data using an OLS parameter estimation.Full-cage population counts are indicated with x-markers and the estimated exponential growth for each cage is plotted with a solid green line. Exponential growth at rate $\bar{r}$, the average across the six replicates, is plotted with a dashed black line.(TIF)

S2 FigNormalized errors induced by subsampling (${\hat{\epsilon }}^{n}$) for synthetic cages generated from tiller counts in category 1.We take *n* = 30, 45, 60 (1-in-3, 1-in-2, and 2-in-3 subsampling strategies, respectively).(TIF)

S3 FigNormalized errors induced by subsampling (${\hat{\epsilon }}^{n}$) for synthetic cages generated from tiller counts in category 2.We take *n* = 30, 45, 60 (1-in-3, 1-in-2, and 2-in-3 subsampling strategies, respectively).(TIF)

S4 FigNormalized errors induced by subsampling (${\hat{\epsilon }}^{n}$) for synthetic cages generated from tiller counts in category 3.We take *n* = 30, 45, 60 (1-in-3, 1-in-2, and 2-in-3 subsampling strategies, respectively).(TIF)
