# Supplementary material for: From theory to experimental design—Quantifying a trait-based theory of predator-prey dynamics
Source: PLoS One. 2018 Apr 25;13(4):e0195919. doi: 10.1371/journal.pone.0195919 (PMC5918917; doi:10.1371/journal.pone.0195919)
Supplement: S1 Appendix — (PDF) [file pone.0195919.s001.pdf]

The parameter μ_{ij} can be interpreted as a measure of similarity in spatial distribution between species in our system. To motivate the mathematical formulation of this parameter, we describe its formulation for species in our experiment, using preliminary observations of habitat use in the greenhouse cages. In S1 Table 1, we state the assumed probability that each species will use a particular region of the cage. We obtained these probabilities by observing the cages on multiple occasions and calculating the fraction of observations where the species was within each region out of all observations.

S1 Table 1. Probability that species used in the cage experiments occupy various regions of the cage.

	Plant	Netting	Ground
A. pisum	0.9	0	0.1
R. padi	0.6	0	0.4
Bembidion	0.05	0.2	0.75
Coccinella	0.5	0.25	0.25
Orius	0.5	0.25	0.25
Pardosa	0.05	0.35	0.6

We split habitat use into the categories "plant," "netting," and "ground." We note that the "ground" region includes the base of the plants, which can be reached by ground-dwelling predators without climbing plants

Recall that for calculation of the overlap parameter we must first define the set Ω of all modes of habitat use and the probability measure μ_i that a species utilizes some habitat within that set. For this example, we would have $\Omega = \{P, N, G, PN, PG, NG, PNG\}$ where P indicates use of the plant region, N indicates use of the netting region, and G indicates use of the ground region. We then have PN indicating use of the plant *and* netting regions, PG indicating use of the plant *and* ground regions, and so forth.

We next define the probability measures μ_i for each species i for habitat use within the set Ω. We state, for example, the definition of μ_A (for species *A. pisum*) on the set:

$$\begin{aligned} &\mu_A(P) = 0.9, \quad \mu_A(N) = 0, \quad \mu_A(G) = 0.1 \\ &\mu_A(PN) = 0.9, \quad \mu_A(PG) = 1, \quad \mu_A(NG) = 0.1, \quad \mu_A(PNG) = 1 \end{aligned} \tag{1}$$

and, for comparison, we state the definition of μ_B (for species *Bembidion*):

$$\begin{aligned} &\mu_B(P) = 0.05, \quad \mu_B(N) = 0.2, \quad \mu_B(G) = 0.75 \\ &\mu_A(PN) = 0.25, \quad \mu_B(PG) = 0.8, \quad \mu_B(NG) = 0.95, \quad \mu_B(PNG) = 1 \end{aligned} \tag{2}$$

We next compute for all species pairs in the system, the total variation $TV(\mu_i, \mu_j)$ in the habitat use distributions. We recall that the definition of total variation is given by $TV(\mu_i, \mu_j) = \sup_{\mathcal{A} \subset \Omega} |\mu_i(\mathcal{A}) - \mu_j(\mathcal{A})|$ and can be interpreted as dissimilarity between the distributions μ_i and μ_j. To compute this value, we take the difference between $\mu_i(\mathcal{A})$ and $\mu_j(\mathcal{A})$ for all sets $\mathcal{A}$ in Ω (such as P or G). We then find the "supremum" over the absolute value of all these differences; that is, we find the largest difference, regardless of sign. For the example we began above, we note that this largest difference occurs when we look at the sets P or NG, since

$$|\mu_A(P) - \mu_B(P)| = |\mu_A(NG) - \mu_B(NG)| = 0.85, \tag{3}$$

and this is the largest magnitude difference we see over all sets in Ω. Then, $TV(\mu_A, \mu_B) = 0.85$. The total variations for all species pairs in our system are given in S1 Table 2.

S1 Table 2. Total variation $TV(\mu_i, \mu_j)$ for species in the cage experiments.

	A. pisum	*R. padi*	*Bembidion*	*Coccinella*	*Orius*	*Pardosa*
A. pisum	0	0.3	0.85	0.4	0.4	0.85
R. padi	0.3	0	0.55	0.25	0.25	0.55
Bembidion	0.85	0.55	0	0.5	0.5	0.15
Coccinella	0.4	0.25	0.5	0	0	0.45
Orius	0.4	0.25	0.5	0	0	0.45
Pardosa	0.85	0.55	0.15	0.45	0.45	0

We calculate these values using the probability measures for habitat use defined in S1 Table 1

We note that a larger value of $TV(\mu_i, \mu_j)$ indicates a higher dissimilarity in habitat use; there exists some mode of habitat use in Ω for which the probability that i uses that region is very different from the probability that j uses that region. Because the values of $\mu_i(\mathcal{A})$ must fall between zero and one (by the definition of a "probability" measure), we will have that all differences between $\mu_i(\mathcal{A})$ and $\mu_j(\mathcal{A})$ fall between zero and one. That is, we will necessarily have $0 \leq TV(\mu_i, \mu_j) \leq 1$ We can therefore see that *A. pisum* and *Bembidion* use the habitat within the cage in a very dissimilar way. However, we see that $TV(\mu_A, \mu_A) = TV(\mu_B, \mu_B) = 0$, since a species will always match its own probability of occupying a given region. We finally note that we will always have $TV(\mu_i, \mu_j) = TV(\mu_j, \mu_i)$; because we take the absolute value of differences in computing the total variation, the order in which we subtract the values of $\mu_i(\mathcal{A})$ and $\mu_j(\mathcal{A})$ does not matter.

The final step in computing habitat use *overlap* is to convert the total variation into a measure of *similarity* instead of dissimilarity. Since we know that $0 \leq TV(\mu_i, \mu_j) \leq 1$, subtracting this quantity from 1 will give us a number that is *still* between 0 and 1. However, the new value is smaller for larger total variations (that is, closer to zero when there is high variation in habitat use) and larger for smaller total variations (closer to one for very similar habitat use). We introduce some scaling factor $0 \leq v_0 \leq 1$ to quantify the effect of assumed habitat use on spatial overlap. If $v_0 = 0$, we do not consider the total variation between spatial distributions at all in computing overlap; that is, we assume all species in the system are equally likely to encounter one another, regardless of their assumed spatial distributions. If $v_0 = 1$, we give full weight to the total variation between species distributions. The final formula for computing overlap, as given in the body of the manuscript, is

$$\nu_{ij} = 1 - v_0 TV(\mu_i, \mu_j). \tag{4}$$
