# Supplementary material for: From theory to experimental design—Quantifying a trait-based theory of predator-prey dynamics
Source: PLoS One. 2018 Apr 25;13(4):e0195919. doi: 10.1371/journal.pone.0195919 (PMC5918917; doi:10.1371/journal.pone.0195919)
Supplement: S2 Appendix — (PDF) [file pone.0195919.s002.pdf]

We present the sensitivity equations for an ATN model of general size, assuming that $a_{ij} = 0$ whenever node j does not predate on node i. Despite this, we retain summations over sets $\mathcal{C}_i$ and $\mathcal{R}_j$ for clarity. We recall that for a system given by $\frac{d\mathbf{N}}{dt} = \mathbf{f}(t, \mathbf{N}, \theta)$, we obtain the sensitivities $\mathbf{s}^\theta = \frac{d\mathbf{N}}{d\theta}$ by solving the differential equation

$$\frac{d\mathbf{s}^\theta}{dt} = \frac{\partial \mathbf{f}}{\partial \mathbf{N}}\mathbf{s}^\theta + \frac{\partial \mathbf{f}}{\partial \theta}, \tag{1}$$

with initial condition $\mathbf{s}^\theta(0) = \mathbf{0}$. For $\mathbf{f}(t, \mathbf{N}, \theta)$ as given by the ATN model and any i, k, we have

$$\begin{aligned} \frac{\partial f_i}{\partial N_k} &= r_i \frac{\partial N_i}{\partial N_k} - \sum_{j \in \mathcal{C}_i} a_{ij} \left(\frac{\partial N_i}{\partial N_k} N_j F_j + N_i \frac{\partial N_j}{\partial N_k} F_j + N_i N_j \frac{\partial F_j}{\partial N_k} \right), \\ \frac{\partial F_j}{\partial N_k} &= -F_j^2 \left(a_{kj} h_{kj} + b_0 a_{jk} \right). \end{aligned} \tag{2}$$

The derivative of f_i with respect to θ is given by

$$\frac{\partial f_i}{\partial \theta} = -\sum_{j \in \mathcal{C}_i} N_i N_j \left(\frac{\partial a_{ij}}{\partial \theta} F_j + a_{ij} \frac{\partial F_j}{\partial \theta} \right), \tag{3}$$

where

$$\begin{aligned} \frac{\partial F_j}{\partial \theta_a} &= -F_j^2 \left(\sum_{k \in \mathcal{R}_j} \frac{\partial a_{kj}}{\partial \theta_a} h_{kj} N_k + \sum_{h \in \mathcal{C}_j} b_0 \frac{\partial a_{jh}}{\partial \theta_a} N_h \right) \quad \text{for } \theta_a = a_0, R_{opt}, \phi, v_0, \\ \frac{\partial F_j}{\partial b_0} &= -F_j^2 \sum_{h \in \mathcal{C}_j} a_{jh} N_h, \\ \frac{\partial F_j}{\partial h_0} &= -F_j^2 \sum_{k \in \mathcal{R}_j} a_{kj} \frac{h_{kj}}{h_0} N_k. \end{aligned} \tag{4}$$

The derivatives of our allometric parameters with respect to a_0, R_{opt}, ϕ, W_i for $i \neq j$ are given by

$$\begin{aligned} \frac{\partial a_{ij}}{\partial a_0} &= \frac{a_{ij}}{a_0}, \\ \frac{\partial a_{ij}}{\partial v_0} &= -a_0 W_i^{1/4} W_j^{1/4} \left(\frac{W_j/W_i}{R_{opt}} e^{1 - \frac{W_j/W_i}{R_{opt}}} \right)^\phi TV(\mu_i, \mu_j), \\ \frac{\partial a_{ij}}{\partial R_{opt}} &= a_{ij} \frac{\phi}{R_{opt}^2} \left(W_j/W_i - R_{opt} \right), \\ \frac{\partial a_{ij}}{\partial \phi} &= a_{ij} \ln \left(\frac{W_j/W_i}{R_{opt}} e^{1 - \frac{W_j/W_i}{R_{opt}}} \right) = a_{ij} \left(1 - \frac{W_j/W_i}{R_{opt}} + \ln \frac{W_j/W_i}{R_{opt}} \right). \end{aligned} \tag{5}$$
