# Supplementary material for: From theory to experimental design—Quantifying a trait-based theory of predator-prey dynamics
Source: PLoS One. 2018 Apr 25;13(4):e0195919. doi: 10.1371/journal.pone.0195919 (PMC5918917; doi:10.1371/journal.pone.0195919)
Supplement: S3 Appendix — (PDF) [file pone.0195919.s003.pdf]

The data utilized in these analyses are from a previous experiment [1] and can be accessed at the Dryad repository https://doi.org/10.5061/dryad.50d1t3p.

We assume exponential growth in predator-free control cages of *R. padi*, with the model solution given by $f(t, r) = 150e^{r_0(t+1)}$, assuming that $t = 0$ one day after aphids are introduced to the cage. Because we only consider aphid population, we temporarily suppress the species indexing and let N denote the aphid population and N_j the sampled population at some time t_j. In current experiments, the protocol is to obtain some estimate r to the growth rate r_0 in predator-free control cages, which is in turn used for the full ATN model in predator-treated cages. We estimate this parameter by seeking the ordinary least squares minimization,

$$r = \arg\min_{r \geq 0} \sum_{j=1}^{M} (N_j - f(t_j, r))^2. \tag{1}$$

If we instead use the subsampled approximations to the population, N_j^n, in the least squares formulation, we obtain the estimate

$$r^n = \arg\min_{r \geq 0} \sum_{j=1}^{M} (N_j^n - f(t_j, r))^2. \tag{2}$$

To investigate the effect of subsampling on the estimated growth rate, we generate synthetic data with a known growth rate and compare the error in parameter estimation under a subsampling scheme for different values of n.

We begin with the control cage data for *R. padi* obtained in a prior microcosm experiment. The provided data includes the per-tiller aphid counts, y_j^k, for six cages, collected on days $t_j = 4, 8$. For each cage, we estimate the growth rate r from the fully sampled population counts, N_j. The resulting parameter estimates for the six cages are given in S3 Table 1 and the model trajectories are plotted in S1 Fig.

S3 Table 1. Estimated growth rates r for each of the six replicate control cages. The average across all growth rates is $\overline{r} = 0.4145$, with an average value of $|r - \overline{r}| = 0.0138$.

Cage	Cage 1	Cage 2	Cage 3	Cage 4	Cage 5	Cage 6	Mean
r	0.4204	0.4045	0.3830	0.4233	0.4233	0.4323	0.4145
$\lvert r - \overline{r} \rvert$	0.0059	0.0099	0.0315	0.0088	0.0088	0.0178	0.0138

For a cage with growth rate r, we compute the normalized difference from model output, ϵ_j^k, at time t_j for each tiller k. Assuming that aphids are uniformly distributed across the cage's T tillers, we have

$$\epsilon_j^k = \frac{1}{f(t_j, r)/T} \left(y_j^k - \frac{f(t_j, r)}{T} \right). \tag{3}$$

For $\overline{r}$ the average of the estimated growth rates across all six cages, we compute the true population curve $f(t, \overline{r}) = 150e^{\overline{r}(t+1)}$. We generate synthetic cages with $T = 90$ tillers which have true population $f(t, \overline{r})/T$ and have been sampled on days $t_j = 4, 8$. We randomly draw observational noise for each tiller from the set of all normalized errors ϵ_j^k for a fixed j.

For each synthetic cage, we consider subsampling strategies in which $n = 5, 6, ..., 90$ tillers are counted. For each strategy, we estimate the growth rate r^n from the approximated population N_j^n. We compute the resulting error $|r^n - \overline{r}|$ for all n, and repeat this process for 200 synthetic cages. We discuss the results of this in the main text, where we plot the average error $|r^n - \overline{r}|$ for each subsampling strategy in Fig 4.

To investigate the effect of subsampling on parameter estimation for the ATN model, we first categorize available mesocosm data by full-cage population values. For all replicate cages of a single treatment and fixed times t_j, we have $N_j \in [0, 100]$, $N_j \in [150, 1500]$, or $N_j \in [4000, 9000]$. We define the set of all tiller counts y_j^k which correspond to full-cage population values in the range of $N_j \in [0, 100]$ as "Category 1," the set of all tiller counts y_j^k which correspond to full-cage population values in the range of $N_j \in [150, 1500]$ as "Category 2," and the set of all tiller counts y_j^k which correspond to full-cage population values in the range of $N_j \in [4000, 9000]$ as "Category 3;" we do not make any distinctions between tiller counts based on the treatment from which they originated or time at which they were obtained

We generate synthetic cages for a given category by randomly sampling 90 tiller counts, $\{\hat{y}^k\}$, from the category. We compute the true population of the synthetic cage,

$$\hat{N} = \sum_{k=1}^{90} \hat{y}^k, \tag{4}$$

and a subsampled approximation to the true population,

$$\hat{N}^n = \sum_{k=1}^{n} \hat{y}^k, \tag{5}$$

for $n = 30, 45$, and 60. We then compute the normalized error induced by subsampling,

$$\hat{\epsilon}^n = \frac{1}{\hat{N}}(\hat{N}^n - \hat{N}), \tag{6}$$

and repeat this process for 1000 synthetic cages. From the synthetic cages, we attempt to identify the distribution of errors $\hat{\epsilon}^n$ for each category. In S2 Fig, S3 Fig, and S4 Fig, we present the scatter plots and histograms for the normalized errors induced by the three subsampling procedures for Categories 1, 2, and 3 respectively.

We note that the distribution of $\hat{\epsilon}^n$ is close to normal for cages in Categories 2 and 3. The variance of the distribution in a given category is higher at lower subsampling rates, and the variance of distributions in Category 2 for a given n are higher than for the corresponding distributions in Category 3. We therefore conclude that for full-cage populations greater than 150 aphids, the normalized error induced by subsampling n tillers is normally distributed, with variance decreasing as the aphid population increases.

As discussed in the main text, we cannot identify a distribution for the $\hat{\epsilon}^n$ for cages in Category 1. If there is a single aphid in a cage and we subsample n of T tillers, then the possible approximations to the population are $\hat{N}^n = T/n, 1$, or 0. Because of this, there is a clear bias towards the upper and lower limits of $\hat{\epsilon}^n$ when the aphid population is low, and we cannot choose a simple distribution to describe the error induced by subsampling in Category 1.

References

1. Jonsson T, Kaartinen R, Jonsson M, Bommarco R. Predictive power of food web models based on body size decreases with trophic complexity; 2018 (to appear - Ecology Letters).
