# Supplementary figures and images for: From theory to experimental design—Quantifying a trait-based theory of predator-prey dynamics
Source: PLoS One. 2018 Apr 25;13(4):e0195919. doi: 10.1371/journal.pone.0195919 (PMC5918917; doi:10.1371/journal.pone.0195919)

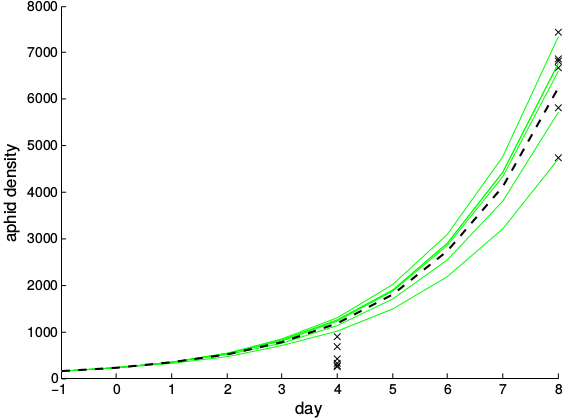

Supplement: S1 Fig — Full-cage population counts are indicated with x-markers and the estimated exponential growth for each cage is plotted with a solid green line. Exponential growth at rate r¯, the average across the six replicates, is plotted with a dashed black line. (TIF) [file pone.0195919.s004.tif]

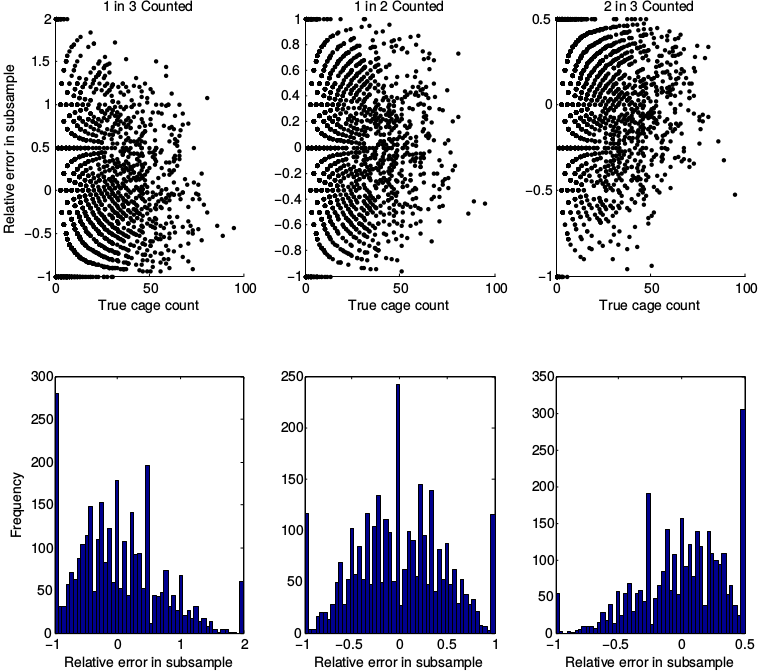

Supplement: S2 Fig — We take n = 30, 45, 60 (1-in-3, 1-in-2, and 2-in-3 subsampling strategies, respectively). (TIF) [file pone.0195919.s005.tif]

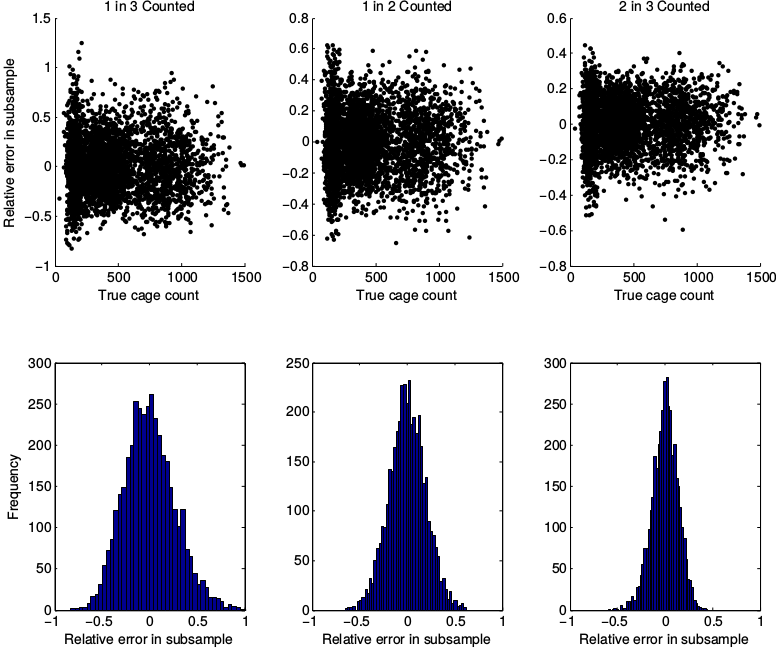

Supplement: S3 Fig — We take n = 30, 45, 60 (1-in-3, 1-in-2, and 2-in-3 subsampling strategies, respectively). (TIF) [file pone.0195919.s006.tif]

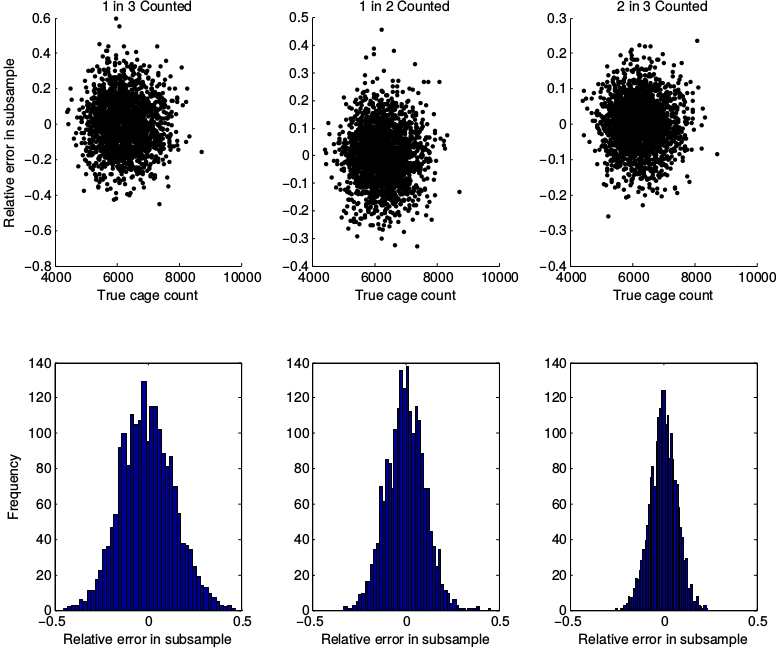

Supplement: S4 Fig — We take n = 30, 45, 60 (1-in-3, 1-in-2, and 2-in-3 subsampling strategies, respectively). (TIF) [file pone.0195919.s007.tif]
